# Identification of Southeast Asian *Anopheles* mosquito species with matrix-assisted laser desorption/ionization time-of-flight mass spectrometry using a cross-correlation approach

**DOI:** 10.1186/s13071-024-06655-1

**Published:** 2025-01-16

**Authors:** Victor Chaumeau, Sunisa Sawasdichai, Thu Zar Ma Ma Moe Min, Thithiwarada Kularbkeeree, Naw Jaruwan, Naw Gloria, Naw Yu Lee, Muesuwa Trackoolchengkaew, Monticha Phanaphadungtham, Patcharamai Rongthong, Aritsara Inta, Wanitda Watthanaworawit, François Nosten

**Affiliations:** 1 Shoklo Malaria Research Unit, Mahidol-Oxford Tropical Medicine Research Unit, Faculty of Tropical Medicine, Mahidol University, Mae Ramat, Thailand; 2 Centre for Tropical Medicine and Global Health, Nuffield Department of Medicine, University of Oxford, Oxford, UK

**Keywords:** Matrix-assisted laser desorption/ionization time-of-flight mass spectrometry, Similarity, Cross-correlation, *Anopheles*, Identification, Sibling species, Southeast Asia, Malaria

## Abstract

**Background:**

Matrix-assisted laser desorption/ionization time-of-flight mass spectrometry (MALDI–TOF MS) is proposed for mosquito species identification. The absence of public repositories sharing mass spectra and open-source data analysis pipelines for fingerprint matching to mosquito species limits the widespread use of this technology. The objective of this study was to develop a free open-source data analysis pipeline for *Anopheles* species identification with MALDI–TOF MS.

**Methods:**

*Anopheles* mosquitoes were captured in 33 villages in Karen (Kayin) state in Myanmar. A subset of 403 specimens was selected for inclusion in either the reference or the test panel (270 and 133 specimens, respectively). Three hundred fifty-nine specimens could be identified with DNA barcodes and were assigned to 21 sensu stricto species and five sibling species pairs or complexes. A total of 3584 mass spectra of the head of these specimens identified with DNA barcoding were acquired and the similarity between mass spectra was quantified using a cross-correlation approach adapted from the published literature. A reference mass spectra database was created using all spectra of the PCR-identified specimens assigned to the reference panel. A simulation experiment was carried out by querying the reference database with the spectra of the test panel to evaluate the performance of species identification with MALDI–TOF MS at varying thresholds of the cross-correlation index for the algorithm to output an identification result and with varying numbers of technical replicates for the tested specimens, considering PCR identification results as the reference.

**Results:**

With one spot and a threshold value of −14 for the cross-correlation index on the log scale, the sensitivity was 0.99 [95% credible interval (CrI): 0.98–1.00], the predictive positive value was 0.99 (95% CrI: 0.98–0.99), and the accuracy was 0.98 (95% CrI: 0.97–0.99). It was not possible to directly estimate the sensitivity and negative predictive value because there was no true negative (i.e., queries of species not referenced in the database) in the assessment.

**Conclusions:**

The cross-correlation approach can be used to match mass spectral fingerprints to predefined taxa. MALDI–TOF MS is a valuable tool for rapid, accurate, and affordable identification of *Anopheles* species.

**Graphical abstract:**

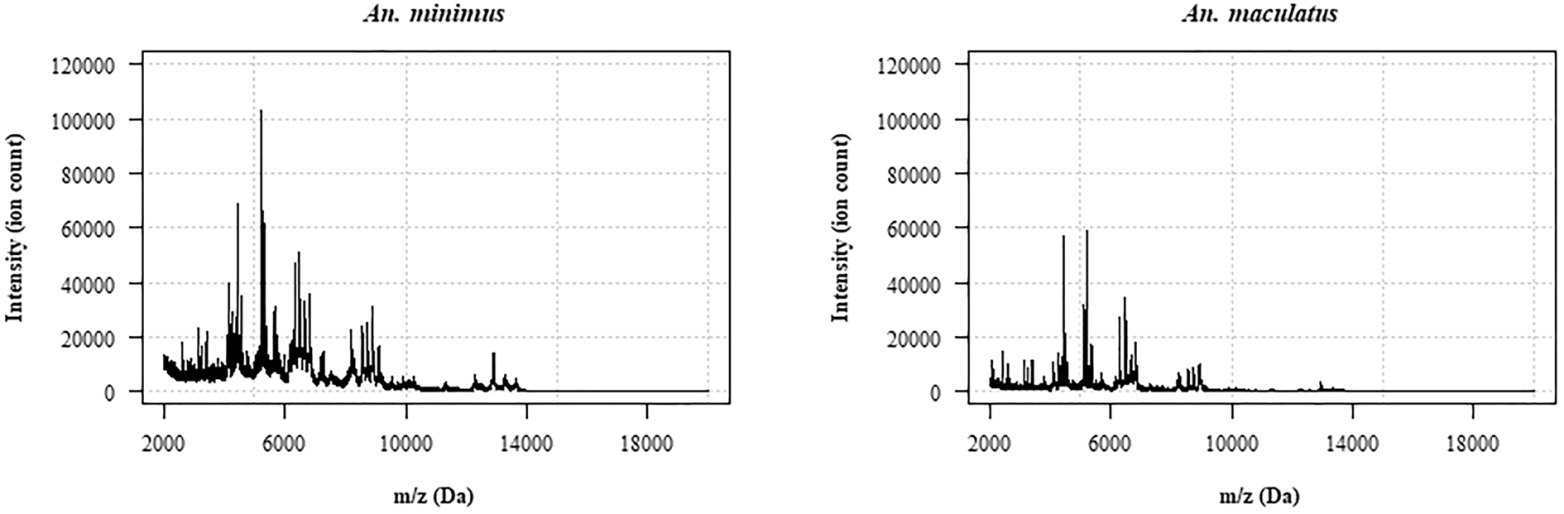

**Supplementary Information:**

The online version contains supplementary material available at 10.1186/s13071-024-06655-1.

## Background

The Thailand–Myanmar border is a rural area of Southeast Asia where people and animals living on both sides of the border are exposed to diseases transmitted by anopheline mosquitoes [1, 2]. Due to specificities in the border ecology, the diversity of mosquito species is among the highest worldwide [3]. Endemic *Anopheles* species are distributed among 18 main groups within the 3 subgenera *Anopheles*, *Baimaia*, and *Cellia* [2]. The main malaria vectors are *An. minimus* (Minimus Complex, Funestus Group), *An. maculatus*, *An. sawadwongporni* (Maculatus Group), *An. dirus*, and *An. baimaii* (Dirus Complex, Leucosphyrus Group). *Anopheles pseudowillmori* (Maculatus Group), *An. aconitus* (Aconitus Subgroup, Funestus Group), and some species of the Annularis and Barbirostris Complexes are secondary vectors. Many other species are suspected vectors because they transmit malaria elsewhere, but their vector status is not well characterized in this region [4, 5]. With regard to lymphatic filariasis transmission, the vector status of *Anopheles* species is not well characterized but many species of the Annularis, Barbirostris, Funestus, Hyrcanus, Leucosphyrus, Maculatus, Subpictus, and Tessellatus Groups were identified as the vectors in neighboring countries [2, 6]. Similarly, there is a knowledge gap on the role of *Anopheles* mosquitoes in arbovirus transmission, but several species have been incriminated as the vectors of some alphaviruses, flaviviruses, orthobunyaviruses, and phleboviruses elsewhere [1, 2]. Accurate vector identification is important to assess transmission dynamics and evaluate vector-control interventions. However, closely related species can have overlapping morphological characters or be completely isomorphic, thereby delimiting cryptic or sibling species complexes that cannot be distinguished by morphology [7].

Matrix-assisted laser desorption/ionization time-of-flight mass spectrometry (MALDI–TOF MS) is used for the identification of living organisms including medically important pathogens and arthropods [8, 9]. Several studies have striven to develop reference mass spectra databases and data analysis methods for *Anopheles* species identification with MALDI–TOF MS [10–14], including Southeast Asian species [15–17]. However, the lack of an open-source data analysis pipeline and sharing of raw mass spectra limit widespread use of this technology. Several methods were proposed to match mass spectral fingerprints of biological specimens to predefined taxa. Signature peaks can be used to distinguish between taxa, but only little information is retained in the analysis and this approach is not reliable with complex matrix and if numerous closely related taxa are to be identified. Alternatively, fingerprint matching can be performed by measuring similarity between mass spectra. Predictive modeling such as linear discriminant analysis or random forest can identify groups of more similar mass spectra corresponding to specimens of the same species [18–20]. This approach has the advantage of not requiring a reference database, but overfitting and the context-specific nature of the similarity metrics they produce can be problematic [21]. Another method is to compute pairwise similarity scores between pairs of mass spectra and to select the spectra pair that has the highest similarity in a query of a reference mass spectra database with a panel of test samples to be identified. This can be implemented with the MALDI Biotyper (Bruker Daltonics, Bremen, Germany) and Vitek system (bioMérieux, Marcy L’Etoile, France) commercial software [8] or with the MSI2 application, a free web application developed at the Sorbonne University for the MALDI–TOF MS identification of medically important fungi, parasites, and arthropods [15, 22–24], but the matching algorithms were not disclosed. Arnold and Reilly have proposed a mathematical algorithm to quantify similarity between MALDI–TO MS mass spectra with a cross-correlation approach and used it to distinguish between 25 strains of *Escherichia coli* [25], but there has been no assessment of this method to identify mosquito species.

The objective of this study was to develop an open-source analysis pipeline for the identification of *Anopheles* species of the Thailand–Myanmar border with MALDI–TOF MS, allowing rapid, accurate, and affordable identification of mosquitoes collected during entomological surveys. *Anopheles* mosquito specimens collected in Karen (Kayin) state in Myanmar were identified with DNA barcodes and analyzed with MALDI–TOF MS. A reference mass spectra database was constructed using open-source software and a cross-correlation algorithm based on previous work by Arnold and Reilly [25]. The performance of identification with MALDI–TOF MS was evaluated using an external dataset generated from specimens captured in the same area. Eventually, the database was upgraded to include all available mass spectra (whether they were initially included in the reference or in the test panel). All analysis code and data are available online, allowing anyone to replicate the methodology and create a reference mass spectra database in house.

## Methods

### Samples collection and preparation

Entomological surveys were carried out in 33 villages in Karen (Kayin) state in Myanmar between 9 November 2020 and 10 October 2022. Mosquitoes were captured into 5 mL plastic tubes using the human-landing catch and animal-baited trap (buffalo, cow, or goat) collection methods. Upon reception in the laboratory (1–10 days after collection), mosquitoes were sorted macroscopically at the genus level and *Anopheles* specimens were identified at the group level using a dichotomic morphological identification key [2]. A subset of these malaria mosquitoes was randomly selected to constitute a panel representative of the different villages and species diversity. Specimens assigned to the reference panel were dissected on the reception day. The dissected head was stored at −80 °C in 1.5 mL microcentrifuge tubes for 85–422 days before being analyzed with MALDI–TOF MS. The remaining anatomical parts were taken into 1.5 mL microcentrifuge tubes containing silica beads and stored at −20 °C for a similar time before being analyzed by PCR. Specimens assigned to the test panel were kept in 1.5 mL microcentrifuge tubes containing silica beads and stored at −20 °C for 392–1069 days before being dissected and analyzed with PCR and MALDI–TOF MS.

### DNA extraction and PCR amplification

DNA was extracted from dissected mosquito abdomens using the cetyltrimethylammonium bromide method as described previously [26]. Amplification of cytochrome c oxidase subunit I (COI) was performed using the primer pair LCO1490 (5′-GGT CAA CAA ATC ATA AAG ATA TTG G-3′) and MTRN (5′-AAA AAT TTT AAT TCC AGT TGG AAC AGC-3′) [27, 28]. The PCR mix was composed of 1× Goldstar DNA polymerase (Eurogentec, Seraing, Belgium, cat. no. PK-0064-02) and 400 nM of each primer. PCR was conducted in a total reaction volume of 25 μL (4 μL of DNA template diluted 1/100 in PCR-grade water and 21 μL of PCR mix). The thermocycling protocol consisted of an initial activation step of 1 min at 94 °C, followed by 40 amplification cycles of 20 s at 94 °C, 20 s at 51 °C, and 30 s at 72 °C. Reactions that failed to amplify the target were repeated with the reverse primer HCO2198 (5′-TAA ACT TCA GGG TGA CCA AAA AAT CA-3′) [27] using the same reaction conditions. Amplification of internal transcribed spacer 2 (ITS-2) was performed using the primer pair ITS2A (5′-TGT GAA CTG CAG GAC ACA T-3′) and ITS2B (5′-ATG CTT AAA TTY AGG GGG T-3′) [29] with the same reaction conditions, except the primer concentration, which was 100 nM each. PCR products were purified using the illustra ExoProStar 1-Step commercial kit (Cytiva, Marlborough, USA, cat. no. US77720) following the manufacturer’s instructions. Sanger sequencing of the purified product was outsourced to Macrogen (Seoul, South Korea) and performed with the forward primer (accession numbers: PP339876-PP340064, PP372871-PP373055, PP968979-PP969128, and PP976491-PP976529). Samples that could not be sequenced were removed from the panel.

### Protein extraction and mass spectra acquisition

Protein extraction and mass spectra acquisition were performed as described previously [15]. Dissected heads were put into 1.5 mL microcentrifuge tubes and rinsed in 70% ethanol for 10 min. The tubes were centrifuged at 18,000 g for 10 min and the supernatant was discarded. After a second centrifugation at 18,000 g for 2 min, the remaining ethanol solution was removed using a micropipette and left to evaporate until dry. Protein extraction was performed by adding 10 μL of 70% formic acid solution (bioMérieux, Lyon, France, cat. no. 411072). After manual homogenization with a micropipette, the homogenates were incubated for 5 min at room temperature. Then, 10 μL of 100% acetonitrile (VWR, Randor, USA, cat. no. 20060.32) was added and the samples were incubated for an additional 5 min. The homogenates were then centrifuged at 18,000 g for 2 min, and 1 μL of the protein extract was deposited onto a disposable target plate (bioMérieux, cat. no. 410893). Once dried, the deposits were covered with 1 μL of alpha-cyano4-hydroxycinnamic acid matrix (bioMérieux, cat. no. 411071). Ten spots were made for each specimen. Mass spectra were acquired with a Vitek system (bioMérieux) in the research use only mode using the Shimadzu Biotech Launchpad MALDI–TOF MS application (Shimadzu Biotech, Kyoto, Japan). The spectra were acquired in linear ion-positive mode at a laser frequency of 60 Hz and a mass range of 2000–20,000 Da. *Escherichia coli* ATCC 8739 was used as a control calibration spot for each run following the manufacturer’s instruction. Raw mass spectra were exported in mzXML file format and used for subsequent analysis.

### *Anopheles* species identification with DNA sequence data

Sanger chromatograms were inspected using Unipro UGENE software version 48 [30] to trim the beginning and the end of each sequence and correct artifactual polymorphisms. ITS-2 was annotated using a 5.8S–28S rRNA interaction and hidden Markov model-based annotation program available online [31]. COI and ITS-2 sequences were queried against the NCBI Nucleotide collection (nr/nt) database using BLASTn [32] and matched to a mosquito species when the identity between the query and subject sequences was ≥ 98%. COI sequences were also queried against the Barcode of Life Data System (BLOD), which includes a larger collection of COI sequences than the NCBI Nucleotide database [33]. To further validate BLAST and BOLD identification results, a phylogenetic analysis was conducted using the study sequences and reference sequences sourced from Genbank. COI sequences were aligned with Clustal W version 2.1 [34] and ITS-2 sequences were aligned with MAFFT using the X-INS-i algorithm and default parameters [35]. The phylogenetic analysis was performed in MrBayes v3.2 [36]. Two models were fitted to the sequence data: a general time-reversible substitution model with gamma-distributed rate variation across sites (GTR + G) and general time-reversible substitution model with gamma-distributed rate variation across sites and a proportion of invariable sites (GTR + G + I). These models were chosen because they are widely used for analysis of mosquito ITS-2 and COI sequence data [37–39]. In the analysis of COI sequences, the dataset was partitioned to estimate different mutation rates for the two first and the third codon positions. Each analysis comprised two independent runs with four chains running for 1,000,000 generations with a sample frequency of 100 generations. The first 25% trees were discarded as burn-in, and posterior probabilities were estimated from the remaining trees to infer branch support. Mixing and convergence of the chains was assessed by examining the average standard deviation of the split frequencies, the traceplot of the loglikelihood, the effective sample size (ESS), and the potential scale reduction factor (PSRF). The best model fit was selected by comparing the marginal model likelihoods (Bayes factor test) [40].

### Construction of the reference mass spectra database

**Fig. 1:**
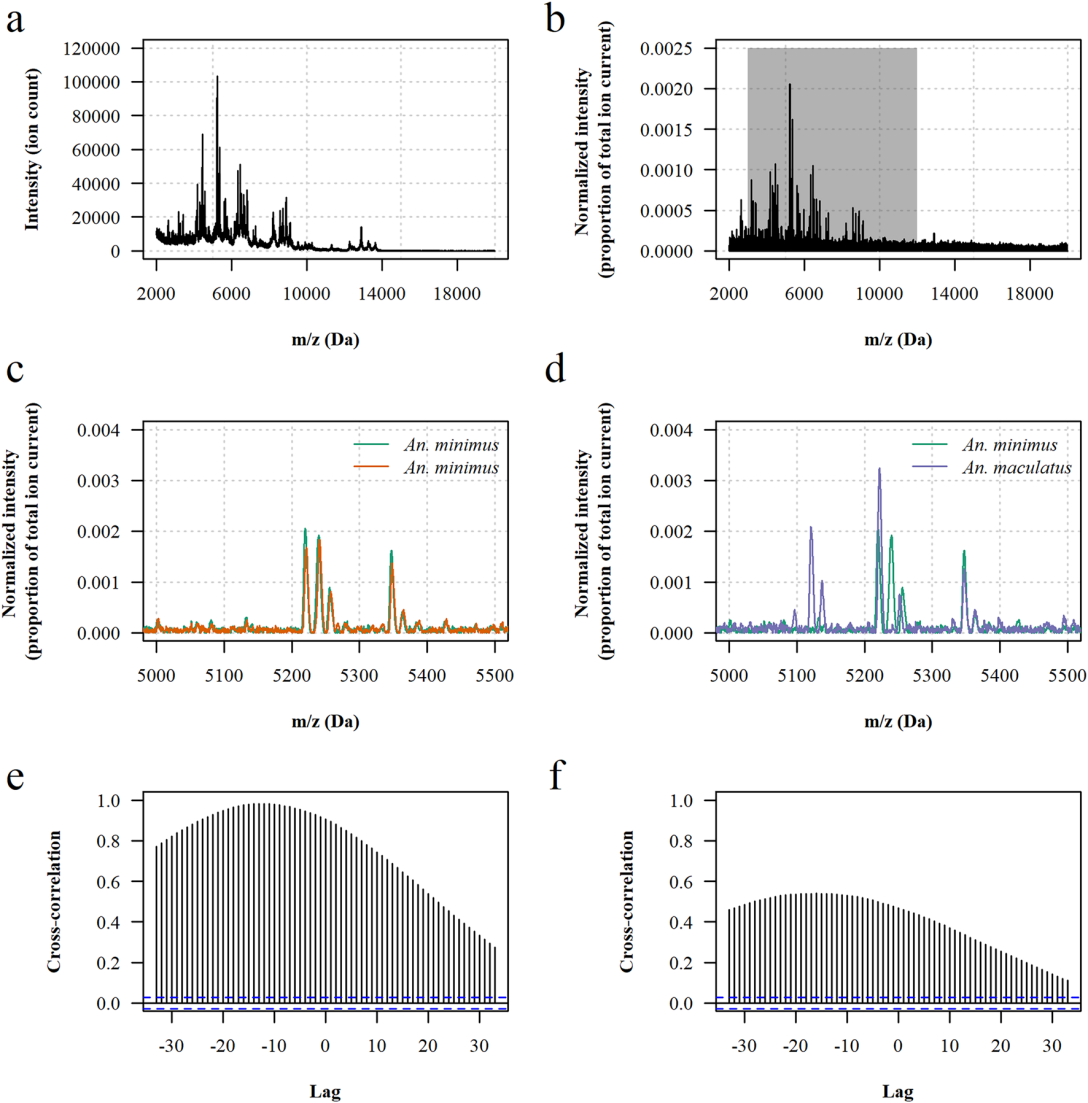
Principle of the cross-correlation algorithm. **a** A typical raw MALDI–TOF mass spectrum of *An. minimus*. **b** The corresponding processed spectrum after intensity normalization and baseline removal. **c** Comparison of the processed mass spectra of two *An. minimus* specimens over the 5000–5500 Da mass interval showing high similarity between spectra. **d** Comparison of the processed mass spectra of an *An. minimus* specimen and of an *An. maculatus* specimen over the 5000–5500 Da mass interval showing limited similarity between spectra. **e** The cross-correlation function of the two *An. minimus* spectra over the 5000–5500 Da mass interval gives a local maximum of 0.982. **f** The cross-correlation function of the *An. minimus* and *An. maculatus* spectra over the 5000–5500 Da mass interval gives a local maximum of 0.540. If no local maximum of the cross-correlation function is detected, the algorithm is parameterized to return 0. The resulting cross-correlation index on the log scale [log_10_(CCI)] over the 3000–12,000 Da mass range is −4.9 for the two *An. minimus* spectra and –infinite for the *An. minimus* and *An. maculatus* spectra

Spectra were visualized and processed using R software version 4.2 and the MALDIquant package version 1.22 [41, 42]. Processing of raw mass spectra included intensity transformation and smoothing, baseline removal, normalization of intensity values and spectra alignment (Fig. 1). A cross-correlation index (CCI) was then calculated for each pairwise spectra comparison using a custom algorithm adapted from previous work [25]. The algorithm outputs the maximum value of the cross-correlation function for two input mass spectra over specified mass intervals, and the CCI is given by the product of the local cross-correlation value of each mass interval. If no local maximum is detected on a given mass interval, the algorithm returns zero. Therefore, the range of possible CCI values on the log scale [log_10_(CCI)] is a real number varying between negative infinite (too dissimilar spectra) and 0 (identical spectra). In this assessment, the CCI algorithm was parameterized with mass intervals of 500 Da spanning the mass range 3000–12,000 Da. This choice was based on previous work by Arnold and Reilly showing that including a number of intervals in the assessment increases the ability of the CCI to discriminate between dissimilar spectra, and that each interval should have a number of discriminative peaks to avoid one interval having a disproportionate weight in the overall metric value [25]. Several mass ranges and mass intervals were tested and the mass range and intervals that gave the best results were used in this analysis (data not shown).

### Evaluation of the performance of MALDI–TOF MS identification

To evaluate the performance of *Anopheles* species identification with MALDI–TOF MS, the test panel was queried against the reference mass spectra database. A simulation experiment was carried out by selecting at random a specified number of spots per specimen of the test panel varying from one to nine with 1000 repeats (few specimens had only nine spectra because one spot failed, the missing values precluded performance evaluation with ten spots). The MALDI–TOF identification result was defined as the reference spectrum giving the highest CCI value considering all randomly selected spots in the analysis. An identification threshold was set for the CCI value below which no identification result was given. The MALDI–TOF identification result was categorized as true positive (test specimen matching with the same species in the reference mass spectra database with a CCI value above the identification threshold), true negative (specimen of a species not represented in the reference mass spectra database with a CCI value below the identification threshold), false positive (matches between different species with a CCI value above the identification threshold), and false negative (species represented in the reference mass spectra database with a CCI value below the identification threshold), considering PCR identification results as the reference. The output of this simulation experiment was used to estimate the sensitivity, positive predictive value and accuracy. Noteworthy, as species coverage in the reference mass spectra database increases with the sample size, very few specimens of species not referenced in the reference mass spectra database are included in the test panel, thereby impairing the estimation of specificity and negative predictive value (because there is no true negative result in the assessment). Therefore, a separate analysis was carried out after excluding comparisons of the same species (i.e., forcing the result to be either falsely positive or truly negative), allowing indirect estimation of the specificity for queries of unreferenced species (i.e., the probability of a result being falsely positive if the query species is not referenced in the reference database), but not of the negative predictive value (because the negative predictive value also depends on the number of false negative).

## Results

### Panel composition

Four hundred three *Anopheles* specimens were selected for inclusion in either the reference or the test panel (270 and 133 specimens, respectively). A total of 254/270 (94%) specimens of the reference panel could be identified based on the analysis of ITS-2 (32 specimens), COI (24 specimens), or both markers (198 specimens). Given the limited added value of COI sequencing for the identification of *Anopheles* species of the reference panel, only ITS-2 sequencing was carried out for the specimens of the test panel and 105/133 (79%) specimens could be identified. For the ITS-2 data, the marginal likelihood supported better fit with the GTR + G model than with the GTR + G + I model (−19,342.79 versus −19,353.13, respectively). For the COI data, the marginal likelihood supported better fit with the GTR + G + I model than with the GTR + G model (−10,781.18 versus −10,835.77, respectively). The resulting phylogenetic trees are presented in Additional file 1: Fig. S1 and Additional file 2: Fig. S2. Based on these DNA sequence data, 359/403 (89%) specimens were assigned to 26 taxa including 21 sensu stricto species and five sibling species pairs or complexes, and were subsequently analyzed with MALDI–TOF MS (Table 1). Two specimens identified as *An. baimaii*/*dirus* based on COI sequencing were not included in the above figures because other specimens of *An. dirus* and *An. baimaii* were identified based on ITS-2 sequencing. Four specimens of the Barbirostris group for which only a portion of the ITS-2 could be sequenced were also removed because the query cover in the BLAST analysis was too short for reliable species identification (approximately 50 base pairs). One specimen of *An. annularis* s.l. had a COI sequence with 97.9% identity with available *An. annularis* s.l. COI DNA barcodes (below the identification threshold of BOLD system) and branched separately from other *An. annularis* s.l. specimens in the phylogenetic tree. Therefore, it was identified as a putatively new sibling species in this complex based on this marker. Similarly, two clades of *An. minimus* were identified based on COI DNA sequences. No distinction was made between these divergent *An. annularis* s.l. and *An. minimus* lineages in subsequent analyses. This choice was based on the results of a subanalysis showing no significant difference between mass spectra of these closely related taxa, as assessed with the matching algorithm used in this study (data not shown).

**Table 1:** Panel composition

Subgenus	Group	Species	No. of specimens in the reference panel	No. of specimens in the test panel	Overall
*Anopheles*	Asiaticus	*An. interruptus*	1	1	2
*Anopheles*	Barbirostris	*An. campestris*/*wejchoochotei*	1	0	1
*Anopheles*	Barbirostris	*An. dissidens*	24	0	24
*Anopheles*	Barbirostris	*An. saeungae*	4	0	4
*Anopheles*	Hyrcanus	*An. peditaeniatus*	3	2	5
*Anopheles*	Hyrcanus	*An. sinensis*	17	2	19
*Cellia*	Annularis	*An. annularis* s.l	10	6	16
*Cellia*	Annularis	*An. nivipes*	13	3	16
*Cellia*	Annularis	*An. philippinensis*	4	1	5
*Cellia*	Funestus	*An. aconitus* s.l	2	6	8
*Cellia*	Funestus	*An. culicifacies* s.l	14	5	19
*Cellia*	Funestus	*An. jeyporiensis*	6	9	15
*Cellia*	Funestus	*An. minimus*	40	11	51
*Cellia*	Funestus	*An. varuna*	2	0	2
*Cellia*	Jamesii	*An. jamesii*	15	2	17
*Cellia*	Jamesii	*An. splendidus*	11	7	18
*Cellia*	Kochi	*An. kochi*	24	7	31
*Cellia*	Leucosphyrus	*An. baimaii*	12	6	18
*Cellia*	Leucosphyrus	*An. dirus*	0	2	2
*Cellia*	Maculatus	*An. dravidicus*	3	2	5
*Cellia*	Maculatus	*An. maculatus*	16	3	19
*Cellia*	Maculatus	*An. pseudowillmori*	10	3	13
*Cellia*	Maculatus	*An. sawadwongporni*	5	2	7
*Cellia*	Subpictus	*An. vagus*	13	8	21
*Cellia*	Tessellatus	*An. tessellatus* s.l	2	9	11
*Cellia*	Unclassified	*An. karwari*	2	8	10

### Construction of the reference mass spectra database

**Fig. 2:**
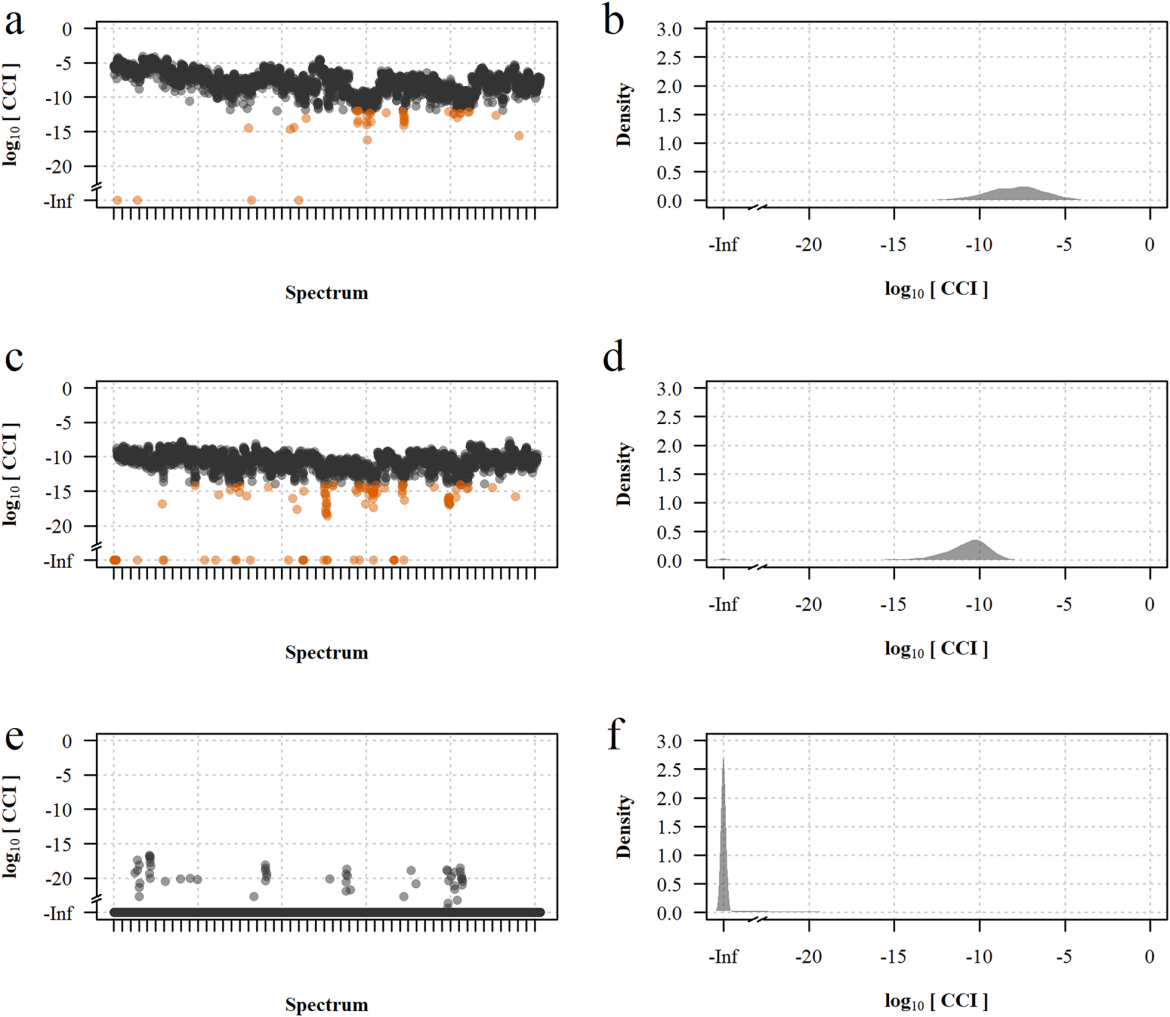
Repeatability, reproducibility and specificity of the mass spectra. **a** Median log_10_(CCI) of pairwise comparisons between technical replicates of the same specimen collated by mass spectra; **b** the corresponding density function. **c** Median log_10_(CCI) of pairwise comparisons between spectra of different specimens of the same species collated by mass spectra; **d** the corresponding density function. **e** Median log_10_(CCI) of pairwise comparisons between spectra of different species collated by mass spectra; **f** the corresponding density function

**Fig. 3:**
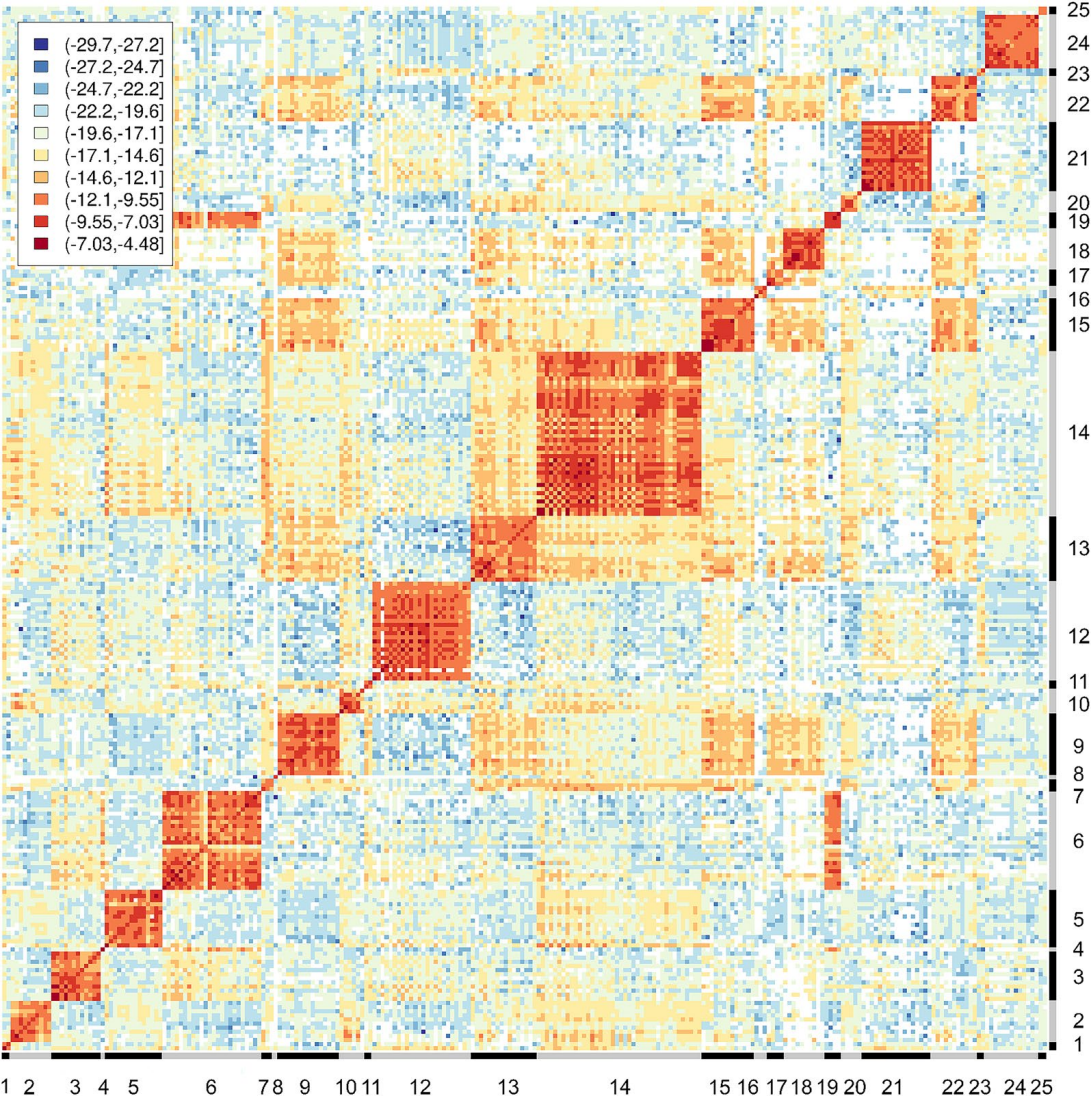
Heat map grid of the median cross-correlation index collated by specimen included in the reference mass spectra database. The red color on the central diagonal and the orange color around the central diagonal show the high repeatability and reproducibility of mass spectra, respectively. The blue color out of the central diagonal shows the high specificity of mass spectra. The orange color out of the central diagonal shows the high similarity between sibling species of the Barbirostris Complex and some species of the Neomyzomyia Series. Negative infinite values are shown in white. 1: *An. aconitus* s.l.; 2: *An. annularis* s.l.; 3: *An. baimaii*; 4: *An. campestris*/*wejchoochotei*; 5: *An. culicifacies* s.l.; 6: *An. dissidens*; 7: *An. dravidicus*; 8: *An. interruptus*; 9: *An. jamesii*; 10: *An. jeyporiensis*; 11: *An. karwari*; 12: *An. kochi*; 13: *An. maculatus*; 14: *An. minimus*; 15: *An. nivipes*; 16: *An. peditaeniatus*; 17: *An. philippinensis*; 18: *An. pseudowillmori*; 19: *An. saeungae*; 20: *An. sawadwongporni*; 21*: An. sinensis*; 22*: An. splendidus*; 23: *An. tessellatus* s.l.; 24: *An. vagus*; 25: *An. varuna*

**Fig. 4:**
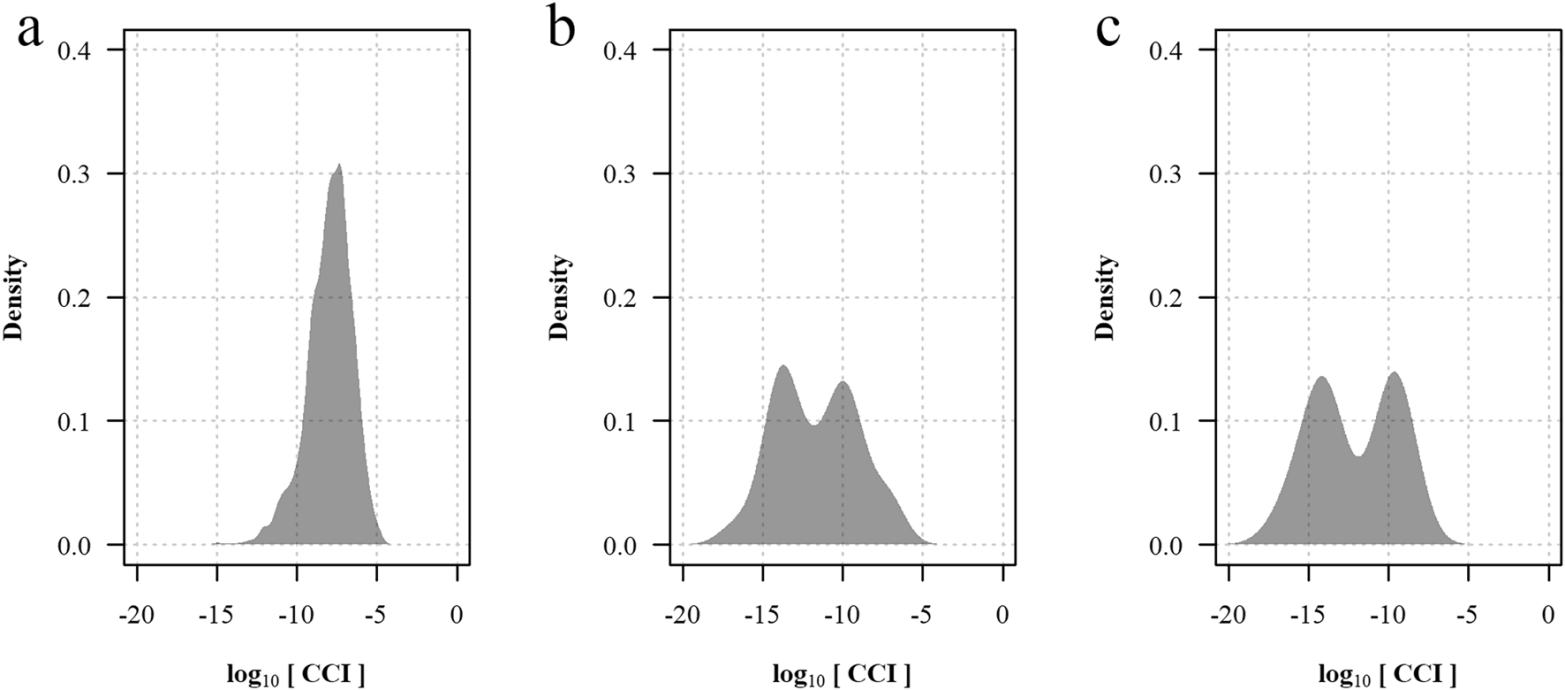
Distribution of the maximum log_10_(CCI) value collated by spectra in a bank-to-bank comparison by the category of result, excluding comparisons between technical replicates of the same sample. **a** Correct matches with another specimen of the same species. **b** True cross-matches between a species referenced in the queried database and a different species. **c** Species represented by only one specimen in the reference mass spectra database and therefore not included in the queried database because self-matching was disabled

A total of 2535 mass spectra of the 254 *Anopheles* specimens selected for inclusion in the reference panel and identified with PCR were acquired, yielding 3,211,845 pairwise comparisons of distinct spectra pairs. The reference mass spectra database covered 25 taxa including 20 sensu stricto species and five sibling species pairs or complexes. A CCI value reflecting similarity between spectra was computed for every spectra pair. The repeatability, reproducibility, and specificity of mass spectra were assessed using subsets of the comparisons (Fig. 2): the median log_10_(CCI) was −7.9 [interquartile range (IQR): −9.2 to −6.8] for comparisons of technical replicates of the same specimen, −10.7 (IQR: −12.6 to −9.4) for comparisons of different specimens of the same species, and –infinite (IQR: –infinite to –infinite) for comparisons of different species. Exclusion of spectra with low repeatability or reproducibility had no significant effect on the performance of MALDI–TOF MS identification (Additional file 3: Table S1), therefore all spectra were kept in subsequent analyses. The heat map grid of the median log_10_(CCI) collated by specimens included in the reference database, despite showing a high similarity between sibling species of the Barbirostris Complex and between some species of the Neomyzomyia Series, confirmed the high repeatability, reproducibility, and specificity of mass spectra (Fig. 3). When considering only the highest CCI value with self-matching disabled, 2477/2535 (97.7%) of the spectra included in the reference database matched with the same species [median log_10_(CCI): −7.8 (IQR: −8.8 to −7.0)] and 58/2535 (2.3%) spectra matched with another species (Fig. 4 and Additional file 4: Table S2). Among the mismatches, 19 were spectra of species represented by only one specimen and thus not included in the queried dataset because self-matching was disabled [median log_10_(CCI): −13.4 (IQR: −14.1 to −9.6)], and 39 were true cross-matches between two referenced species [median log_10_(CCI): −11.7 (IQR: −13.6 to −9.8)]. Interestingly, the distribution of CCI values in concordant matches varied across taxa, suggesting variable level of interspecimen reproducibility and possible cryptic diversity in some taxa (Additional file 5: Table S3). True cross-matches were observed between *An. dissidens* and *An. saeungae* [two spectra, log_10_(CCI): −9.1 and −7.0] and vice versa [five spectra, median log_10_(CCI): −7.0 (IQR: −7.5 to −7.0)], between *An. dravidicus* and *An. maculatus* [four spectra, median log_10_(CCI): −9.7 (IQR: −10.0 to −9.6)], *An. minimus* [one spectrum, log_10_(CCI): −10.0], *An. nivipes* [one spectrum, log_10_(CCI): −10.2], and *An. splendidus* [one spectrum, log_10_(CCI): −9.9], between *An. karwari* and *An. dissidens* [one spectrum, log_10_(CCI): −11.8] and *An. jamesii* [one spectrum, log_10_(CCI): −9.9], between *An. kochi* and *An. pseudowillmori* [one spectrum, log_10_(CCI): −17.0], between *An. pseudowillmori* and *An. maculatus* [two spectra, log_10_(CCI): −11.7 and −11.9], between *An. sawadwongporni* and *An. maculatus* [eight spectra, median log_10_(CCI): −13.8 (IQR: −14.0 to −13.6)] and *An. nivipes* [two spectra, log_10_(CCI): −14.5 and −13.9], and between *An. tessellatus* s.l. and *An. kochi* [10 spectra, median log_10_(CCI): −12.2 (IQR: −13.1 to −11.0)]. Among the species represented by only one specimen, 8/9 *An. campestris*/*wejchoochotei* spectra matched with *An. dissidens* [median log_10_(CCI): −9.4 (IQR: −9.7 to −9.2)] and the remaining spectrum matched with *An. saeungae* [log_10_(CCI) = 10.2], and 10/10 *An. interruptus* spectra matched with *An. minimus* [median log_10_(CCI): −14.1 (IQR: −14.5 to −13.8)].

### Evaluation of the performance

**Fig. 5:**
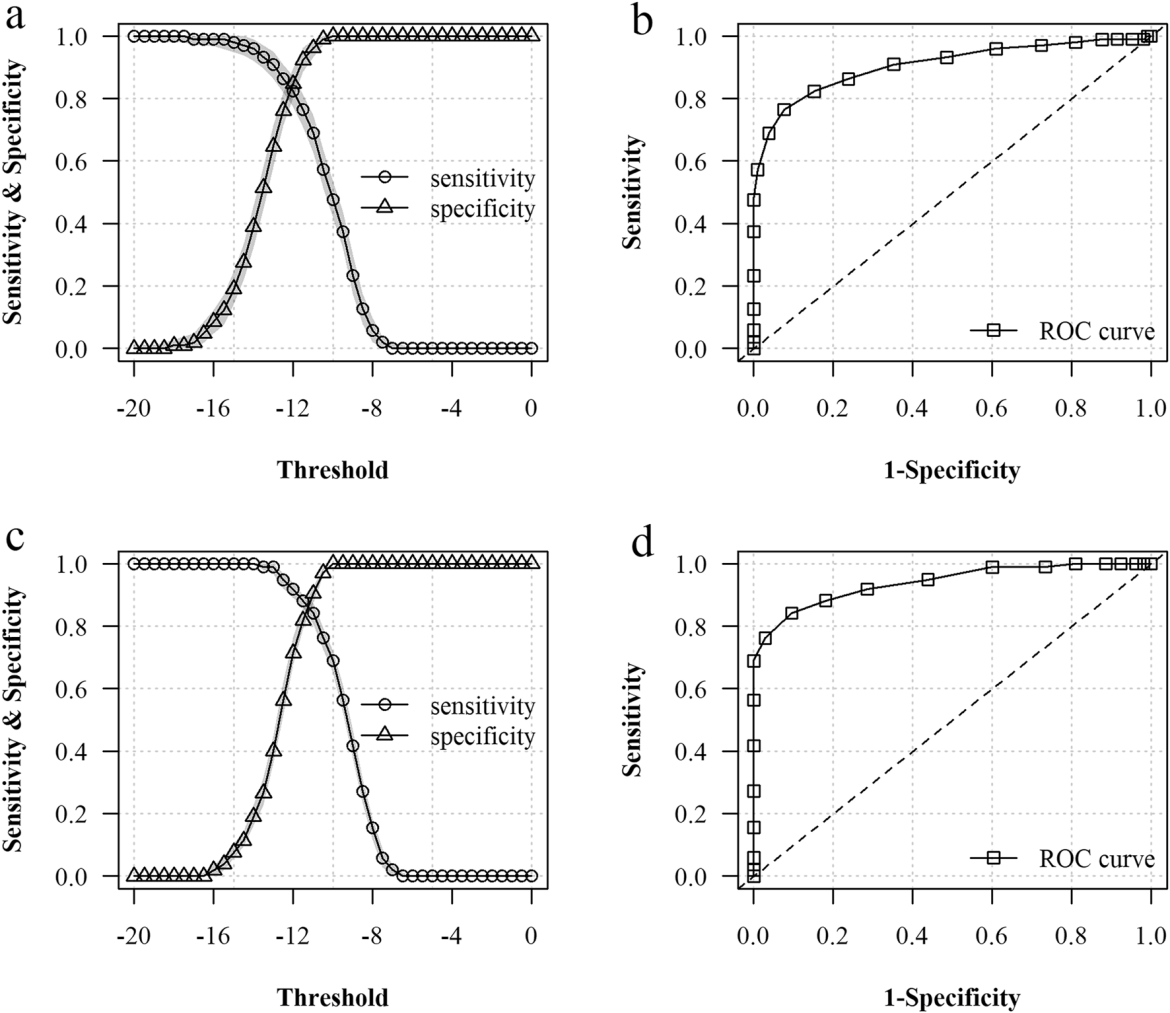
Evaluation of the performance of the reference mass spectra database for *Anopheles* species identification using the test panel. **a** Sensitivity and specificity estimated at varying identification threshold considering one spot per specimen; **b** the corresponding receiving operator characteristics curve. **c** Sensitivity and specificity estimated at varying identification threshold considering four spots per specimen; **d** the corresponding receiving operator characteristics curve. The shaded areas in panels **a** and **c** show the 95% credible interval around the median value of 1000 simulations. The dashed line in panels **b** and **d** shows the performance of a random classification

To evaluate the performance of *Anopheles* species identification with MALDI–TOF MS, 1049 mass spectra of the 105 PCR-identified specimens included in the test panel were queried against the reference database, yielding 2,659,215 pairwise comparisons. The sensitivity, positive predictive value, and accuracy were estimated at varying values of identification thresholds and numbers of technical replicates by carrying out a simulation experiment with 1000 repeats. In this experiment, a subset of the spectra was selected at random for each specimen of the test panel, and the identification result was defined as the reference mass spectra giving the highest CCI value considering all randomly selected replicates for a given test specimen. Notably, true negative results become rare as species coverage increases in the reference database, thus impairing the estimation of the specificity and negative predictive value. Therefore, the specificity was assessed by disabling matches between specimens of the same species, thereby mimicking queries of unreferenced species only and forcing the result to be either truly negative of falsely positive (see Methods). Increasing the number of technical replicates increased the sensitivity and decreased the specificity, resulting in an increased classification accuracy, whereas increasing the identification threshold toward a more stringent value decreased the sensitivity and increased the specificity, resulting in a decreased classification accuracy (Table 2 and Fig. 5). Setting the identification threshold to −14 (low stringency) and considering one spot in the analysis, the sensitivity was 0.96 [95% credible interval (CrI): 0.92–0.99], the specificity was 0.39 (95% CrI: 0.32–0.46), the positive predictive value was 0.94 (95% CrI: 0.92–0.96), and the accuracy was 0.90 (95% CrI: 0.87–0.94). With four spots, the sensitivity was 1.00 (95% CrI: 1.00–1.00), the specificity 0.19 (95% CrI: 0.16–0.23), the positive predictive value 0.93 (95% CrI: 0.91–0.95), and the accuracy 0.93 (95% CrI: 0.92–0.93). When considering an identification threshold of −12 (high stringency) and either one or four spots in the analysis, the sensitivity was 0.82 (95% CrI: 0.77–0.87) versus 0.92 (95% CrI: 0.89–0.94), the specificity 0.85 (95% CrI: 0.80–0.90) versus 0.71 (95% CrI: 0.68–0.76), the positive predictive value was 0.97 (95% CrI: 0.94–0.99) versus 0.95 (95% CrI: 0.94–0.97), and the accuracy was 0.81 (95% CrI: 0.75–0.86) versus 0.88 (95% CrI: 0.85–0.9), respectively. The list of spectra that gave a false positive or false negative result considering an identification threshold of −14 and all simulation repeats is provided in the Additional file 6: Table S4.

**Table 2:** Performance of *Anopheles* species identification with MALDI–TOF MS

Threshold	No. spots	Sensitivity (95% CrI)	Specificity (95% CrI) ^a^	Positive predictive value (95% CrI)	Accuracy (95% CrI)
−14	1	0.96 (0.92–0.99)	0.39 (0.32–0.46)	0.94 (0.92–0.96)	0.9 (0.87–0.94)
−14	2	1 (0.98–1)	0.27 (0.22–0.31)	0.94 (0.91–0.96)	0.93 (0.9–0.96)
−14	4	1 (1–1)	0.19 (0.16–0.23)	0.93 (0.91–0.95)	0.93 (0.91–0.95)
−14	9	1 (1–1)	0.14 (0.14–0.16)	0.92 (0.92–0.93)	0.92 (0.92–0.93)
−13	1	0.91 (0.86–0.95)	0.65 (0.58–0.71)	0.95 (0.93–0.97)	0.87 (0.83–0.9)
−13	2	0.96 (0.93–0.99)	0.51 (0.46–0.58)	0.95 (0.92–0.97)	0.91 (0.88–0.94)
−13	4	0.99 (0.97–1)	0.4 (0.36–0.45)	0.93 (0.91–0.96)	0.92 (0.9–0.95)
−13	9	1 (0.99–1)	0.3 (0.3–0.32)	0.92 (0.92–0.93)	0.92 (0.91–0.93)
−12	1	0.82 (0.77–0.87)	0.85 (0.8–0.9)	0.97 (0.94–0.99)	0.81 (0.75–0.86)
−12	2	0.89 (0.86–0.92)	0.78 (0.73–0.83)	0.96 (0.94–0.98)	0.86 (0.83–0.89)
−12	4	0.92 (0.89–0.94)	0.71 (0.68–0.76)	0.95 (0.94–0.97)	0.88 (0.85–0.9)
−12	9	0.95 (0.93–0.95)	0.62 (0.61–0.65)	0.94 (0.94–0.95)	0.9 (0.88–0.9)

^a^Specificity was assessed with matching between specimens of the same species disabled because there was no true negative in the dataset (i.e., no queries of unreferenced species, see Methods)

### Database upgrade

Finally, the reference database was upgraded to include all PCR-identified specimens (whether they were initially included in the reference or in the test panel) and the same analysis was carried out. The upgraded database was composed of 3584 spectra of 359 specimens including 21 sensu stricto species and five sibling species pairs or complexes (Table 1). In bank-to-bank analysis with self-matching disabled, 3528/3584 (98.4%) of the spectra matched with the same species [median log_10_(CCI): −8.3 (IQR: −9.5 to −7.3)] and 56/3584 (1.6%) with another species [47 were true cross-matches, median log_10_(CCI): −12.4 (IQR: −13.8 to −10.1) and nine were queries of species represented by only one specimen in the upgraded database, median log_10_(CCI): −9.5 (IQR: −10.1 to −9.2), Additional file 7: Table S5]. Setting the identification threshold to −14 and considering one spot in the analysis, the sensitivity was 0.99 (95% CrI: 0.98–1.00), the specificity (for queries of unreferenced species) was 0.18 (95% CrI: 0.16–0.21), the predictive positive value was 0.99 (95% CrI: 0.98–0.99), and the accuracy was 0.98 (95% CrI: 0.97–0.99, Additional file 8: Table S6).

## Discussion

A reference mass spectra database for the identification of *Anopheles* mosquito species of the Thailand–Myanmar border with MALDI–TOF MS was developed. The database references 21 sensu stricto species and five sibling species pairs or complexes, including the main vector species of this area. Fingerprint matching of *Anopheles* species was carried out with a custom algorithm based on the seminal work of Arnold and Reilly, who first described the principle of the cross-correlation index as a quantitative measure of similarity between mass spectra [25]. In this assessment, the identification performances were excellent with a sensitivity of 0.99 (95% CrI: 0.98–1.00), a predictive positive value of 0.99 (95% CrI: 0.98–0.99), and an accuracy of 0.98 (95% CrI: 0.97–0.99) considering an identification threshold value for the log_10_(CCI) of −14 and only one technical replicate per test specimen to be identified. Notably, queries of unreferenced species become rare as species coverage in the reference database increases [43], thus impairing the estimation of specificity and negative predictive value (because there is no true negative in the assessment). By disabling matches between spectra of the same species (i.e., mimicking queries of unreferenced species and forcing the MALDI–TOF MS identification result to be either false positive or true negative), the estimated specificity was only 0.18 (95%CI 0.16–0.21). This finding suggests that using an identification threshold with low stringency could give poor identification performance if the species composition of the test panel is different than that of the reference mass spectra library, and highlights the importance of exhaustive species referencing in the reference mass spectra database or concomitant assessment of species diversity in a subset of the samples to be identified with MALDI–TOF MS with a DNA sequencing approach.

In this setting, about a 100 mosquito specimens can be identified with MADLI–TOF MS per day, for a cost of approximately USD 1.5 per sample. The main factors limiting the throughput are the manual method used to extract proteins and the relatively long acquisition time. Future studies may strive to address these limitations. The main cost driver was the use of disposable slides (30% of the total cost) because there are no commercially available reusable slides for the MALDI–TOF MS device used in this study. This finding highlights the importance of considering the cost of consumables when procuring a MALDI–TOF MS device, as it may vary a lot between device manufacturers and countries. *Anopheles* species identification with MALDI–TOF MS has a higher taxonomic resolution than routine morphology, which can give a reliable identification result at the group or subgroup level for 500 to 800 specimens per person per day with reasonably achievable expertise in entomology. Morphological identification at the species or sibling species complex level requires a level of expertise in mosquito taxonomy that can be difficult to achieve, very good quality specimens, and the throughput is less than for simple classification at the group or subgroup level. MALDI–TOF MS proved more reliable than the PCR assays used in this study: 98% of the PCR-identified specimens were accurately identified with MALDI–TOF MS (considering PCR as the reference) whereas only 89% of the specimens initially selected for inclusion in the panel could be identified with PCR, despite repeated testing. Although the performance of PCR assays may be better in other settings, especially with a well-optimized high-throughput DNA sequencing platform as was recently developed for the identification of mosquito species across the entire *Anopheles* genus [44, 45], this result demonstrates that very good identification performance can be achieved relatively easily with MALDI–TOF MS technology in settings with limited expertise in molecular biology.

This study has several limitations. Species coverage was not exhaustive and some specimens could not be identified at the species level. Therefore, the ability of the CCI algorithm to differentiate between sibling species remains largely undetermined. This limitation could be alleviated by carrying out additional testing of the remaining DNA extracts and by increasing the sampling efforts to reference more species in the database. The performance of the database for the identification of specimens of the same species collected in different areas was not assessed. Additional mosquito collection and acquisition of reference mass spectra (i.e., high-quality mass spectra from molecularly identified specimen) would be required to alleviate this limitation. Other anatomical parts were not assessed. Several studies have striven to evaluate the effects of body part on the performance of species identification with MALDI–TOF MS including mosquitoes [12, 46, 47], sand flies [48], tsetse flies [49], ticks [50], and fleas [51]. For mosquitoes, there is no consensus on the anatomical part that gives the best performance. One study found better results using the head than legs and thorax [12], one study found similar results with the thorax and legs but the head was not assessed [46], and one study found better results with the thorax and legs than with the head [47]. The head was used in this study because it preserves the remaining parts (including the guts and salivary glands) for pathogen screening and mosquito genotyping. Furthermore, the legs are easily lost, especially after exposure to pyrethroid insecticides, and are prone to cross-contamination when specimens are pooled during collection, transport, storage, or handling. Comparisons of different anatomical parts was out of the scope of this study as it would have required a lot of additional testing, but it is a recommendation for future research. The effects of storage conditions and duration on the performance were not assessed. This would have required a different design and was also out of the scope of the current study.

Future research should be carried out to improve the code and develop an identification service readily available to end-users with limited bioinformatics skills, as was previously proposed with the MSI2 application for medically important fungi, parasites, and arthropods [15, 22–24]. Moreover, the performance and added value of *Anopheles* species identification with MALDI–TOF MS should be further validated at scale, and head-to-head comparisons with other identification methods would be valuable. Non-malaria mosquitoes include important vector species. Little is known about their ecology and biology in relation to disease transmission because of the challenges associated with sampling and identification [52–55]. Additional studies should be carried out to develop a reference mass spectra library for the identification of non-malaria mosquito species and, more generally, of arthropod vectors of medical and veterinary importance, to support investigations of the diseases they transmit.

Accurate, fast, and affordable vector species identification is paramount to investigate the transmission dynamics of mosquito-borne diseases and evaluate the impact of control interventions. Despite decades of research, MALDI–TOF MS is seldom used operationally. This is largely due to the lack of an open-source data analysis pipeline and data sharing. This study is an important step forward toward the widespread use of MALDI–TOF MS for the identification of arthropod vectors in resource-limited settings.

## Conclusions

The cross-correlation approach is an efficient analysis framework for fingerprint matching of *Anopheles* mosquito species with MALDI–TOF MS. MALDI–TOF MS is a promising tool for malaria vector identification, but further research is needed to validate it at scale.

## Supplementary Information

**Additional file 1: Fig. S1.** Phylogenetic tree for the COI sequences of the *Anopheles* specimens included in the panel. Reference sequences sourced from GenBank are shown in red.

**Additional file 2: Fig. S2.** Phylogenetic tree for the ITS-2 sequences of the *Anopheles* specimens included in the panel. Reference sequences sourced from GenBank are shown in red.

**Additional file 3: Table S1.** Results of the sensitivity analysis.

**Additional file 4: Table S2.** List of mismatches.

**Additional file 5: Table S3.** Distribution of CCI values in concordant matches.

**Additional file 6: Table S4.** List of wrong identifications.

**Additional file 7: Table S5.** List of mismatches after database upgrade.

**Additional file 8: Table S6.** Results of performance evaluation after database upgrade.

## Data Availability

All analysis code and data are available via an accompanying github repository: https://github.com/victorSMRU/malditof-cci. The dataset was deposited on Zenodo: https://zenodo.org/records/12704052.

## Abbreviations

CCI: Cross-correlation index
COI: Cytochrome c oxidase I
ITS-2: Internal transcribed spacer 2
MALDI–TOF MS: Matrix-assisted laser desorption/ionization time-of-flight mass spectrometry
