# Supplementary figures and images for: Identification of Southeast Asian Anopheles mosquito species with matrix-assisted laser desorption/ionization time-of-flight mass spectrometry using a cross-correlation approach
Source: Parasit Vectors. 2025 Jan 16;18:8. doi: 10.1186/s13071-024-06655-1 (PMC11737280; doi:10.1186/s13071-024-06655-1)

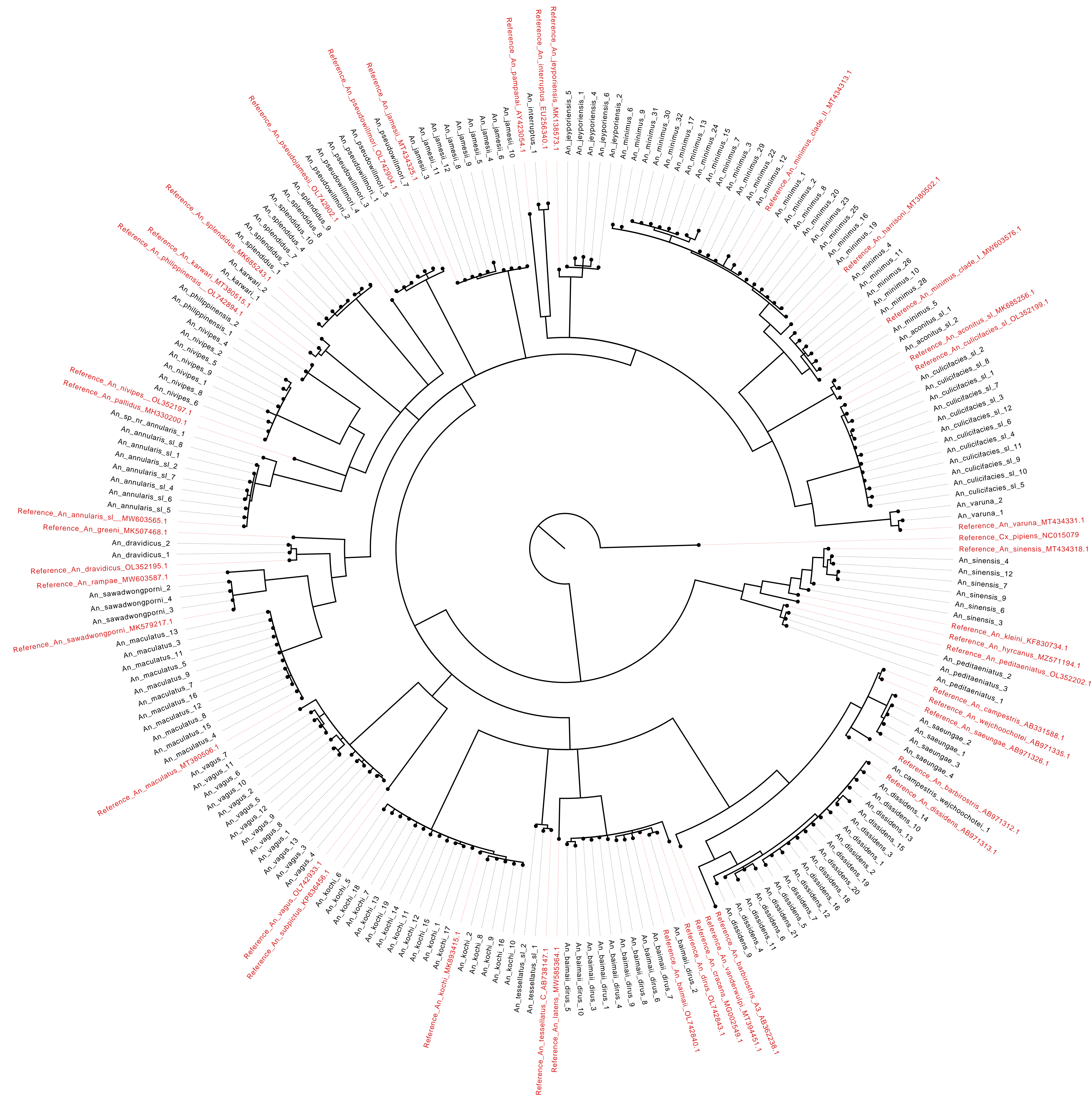

Supplement: Supplementary file 1 — Additional file 1: Fig. S1. Phylogenetic tree for the COI sequences of the Anopheles specimens included in the panel. Reference sequences sourced from GenBank are shown in red. [file 13071_2024_6655_MOESM1_ESM.pdf]

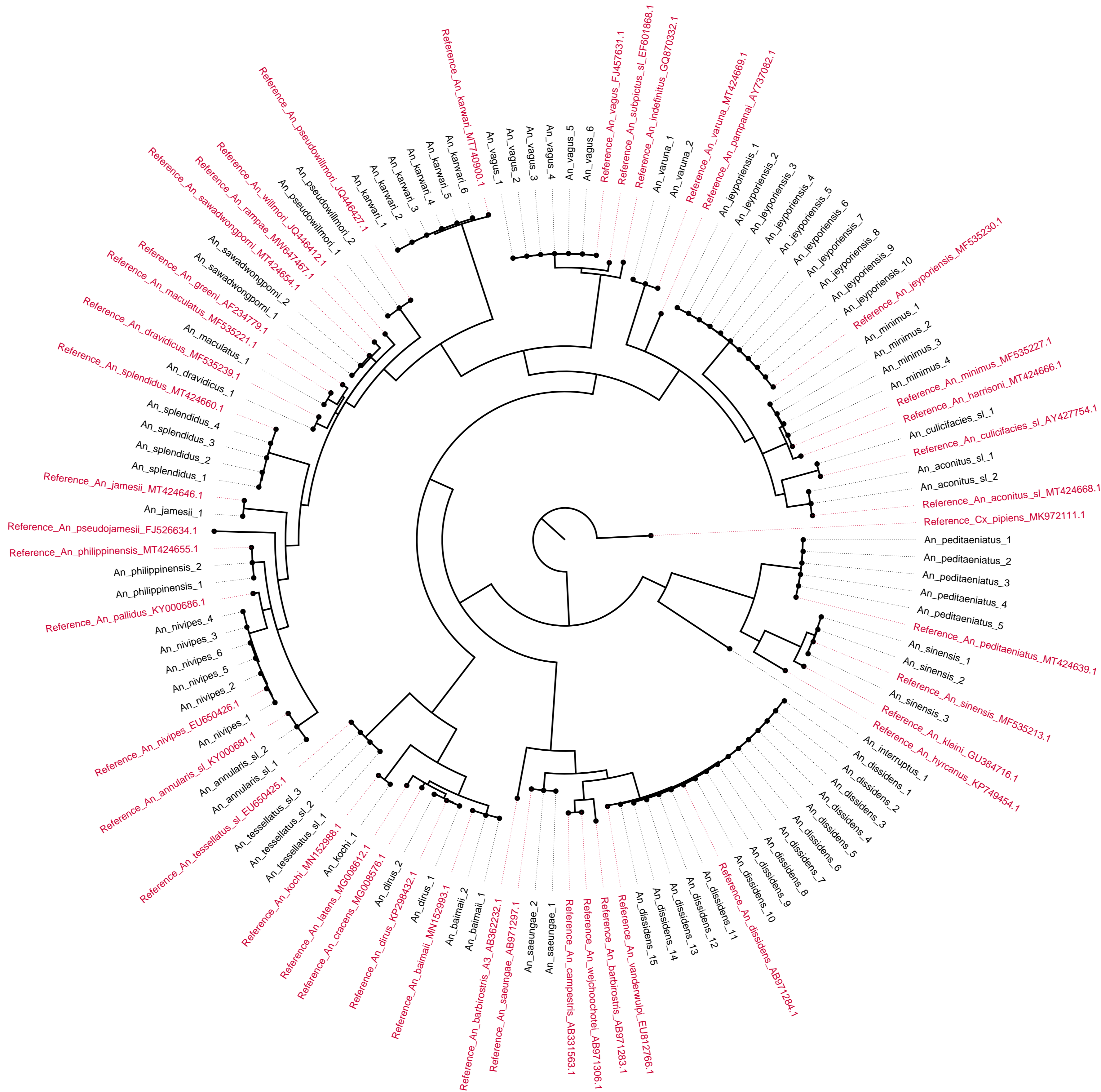

Supplement: Supplementary file 2 — Additional file 2: Fig. S2. Phylogenetic tree for the ITS-2 sequences of the Anopheles specimens included in the panel. Reference sequences sourced from GenBank are shown in red. [file 13071_2024_6655_MOESM2_ESM.pdf]
